# Hemoporfin photodynamic therapy for port-wine stains in children: a prospective study

**DOI:** 10.3389/fmed.2026.1719323

**Published:** 2026-09-01

**Authors:** Jun Cheng, Hanxiang Bai, Hua Yuan, Yun Zou, Mengyu Huang, Zhiping Wu, Ronghua Fu

**Affiliations:** Department of Plastic Surgery, Jiangxi Provincial Children’s Hospital, Nanchang, China

**Keywords:** children, hemoporfin, photodynamic therapy (PDT), port-wine stains (PWS), prospective study

## Abstract

**Introduction:**

Hemoporfin photodynamic therapy is a promising treatment for port-wine stains. However, its efficacy and safety in pediatric cases remain controversial. This study investigated the efficacy and safety of porphyrin photodynamic therapy for treating port-wine stains in children.

**Methods:**

In this prospective study, 140 children with port-wine stains were included and all patients received hemoporfin photodynamic therapy at least twice. Their demographic characteristics, treatment outcomes, and adverse events were recorded.

**Results:**

After two treatment sessions, 79.3% of pediatric patients demonstrated a response. The response rate was significantly higher in patients under 6 years old than those aged 12–18 years. The response rate for the red variant of PWS was significantly higher than that for the purple variant. The response rate in the limbs was markedly lower than that in the face. However, gender, prior PDL treatment history, lesion size exceeding 30 cm^2^, or treatment intervals longer than 6 months did not affect efficacy. All patients tolerated most adverse reactions well.

**Conclusion:**

Hemoporfin photodynamic therapy is an effective and safe treatment for port-wine stains. Patients with the red type, facial involvement, and those under 6 years of age demonstrate superior efficacy.

## Introduction

Port-wine stain (PWS) is a congenital progressive capillary malformation that does not resolve spontaneously, with an incidence of 0.3–0.5% in newborns (1–3). PWS commonly occurs in the head and neck region (3, 4), and lesions tend to grow larger, darker, and thicker with age, potentially developing into hypertrophic or nodular changes (5), which can cause psychological distress for patients and their families (6, 7). Currently, pulsed dye laser (PDL) is the gold standard treatment for PWS (8, 9). However, it is estimated that less than 10% of patients achieve complete clearance after PDL treatment (10).

Vascular-targeted photodynamic therapy (PDT) may be another option. Vascular PDT concentrates photosensitizers in abnormal capillaries and exposes them to light of an appropriate wavelength, generating singlet oxygen to selectively destroy abnormal capillaries (11, 12). Hemoporfin is a novel second-generation porphyrin-type photosensitizer that exhibits stronger photodynamic effects than first-generation photosensitizers and lower skin phototoxicity, enabling repeated photodynamic therapy (PDT) in the short term (13).

Hematoporphyrin monomethyl ether (HMME)-PDT was approved in China in 2017 for the treatment of PWS (14). However, current research on young children is limited, and there is a lack of studies supporting the safety of blood porphyrin use in young children. This study focuses on patients under the age of 18 and aims to conduct a promising analysis of the efficacy and influencing factors of hemoporfin.

## Materials and methods

This study prospectively included PWS patients who underwent HMME-PDT at the Department of Plastic and Aesthetic Surgery of Jiangxi Children’s Hospital between January 2022 and December 2024. The study was approved by the hospital’s Institutional Review Board and conducted in accordance with the Declaration of Helsinki. Appropriate informed consent was obtained from all patients or their parents prior to participation.

Inclusion criteria were as follows: age between 1 and 18 years; clinical diagnosis of port-wine stain; receiving at least two sessions of hemoporfin photodynamic therapy; and complete follow-up data. Exclusion criteria were as follows: age over 18 years; failure to complete follow-up; allergy to hemoporfin; keloid-prone skin; increased sensitivity to light; abnormal liver or kidney function; or prior history of photodynamic or isotope therapy.

All patients were treated under general anesthesia. After carefully covering normal skin, hematoporphyrin monomethyl ether (HMME; Shanghai Fudan-Zhangjiang Bio-Pharmaceutical Co., Ltd., Shanghai, China) was administered intravenously at a dose of 5 mg/kg at a constant rate over 20 min. Ten minutes after infusion, the target PWS lesion was exposed to 532 nm green light from a light-emitting diode (LED) source (Wuhan Yage Photoelectric Technology Co., Ltd., Wuhan Yage LED1) with a power density of 80–95 mW/cm^2^. During irradiation, forced air cooling was applied concurrently to protect the epidermis, maintaining the skin surface temperature between 40–41 °C. Post-treatment, ice packs were applied intermittently for skin cooling over 3 days to minimize pain and potential thermal damage. To prevent photosensitivity reactions, patients were instructed to avoid strong light exposure for at least 14 days after treatment. The interval between repeated treatments was generally 2–3 months. If a patient showed poor response to previous HMME-PDT sessions, the power density was increased by 5 mW/cm^2^ (maximum power density: 95 mW/cm^2^).

Lesions were photographed before treatment and at the 6-month follow-up after the final treatment session, using a fixed light source, fixed positioning, and a consistent camera (EOS 80D; Canon, Tokyo, Japan), with no beauty filters or image retouching applied. Improvement was graded into five categories: None (no difference), Poor (lightening <25%), Fair (25–49%), Good (50–74%), and Excellent (75–100%). Patients with improvement ≥25% were defined as responders. If a patient had multiple lesions, the site for evaluation was selected based on the patient’s (or parents’) primary concern and expectation for improvement. For multiple lesions with similar concern levels, only the largest lesion was evaluated. Treatment efficacy was evaluated by three independent, blinded senior attending or associate chief dermatologists with at least 5 years of clinical experience in photodynamic therapy (PDT). The assessors reviewed the standardized photographs in a randomized order and were denied access to any patient identifiers. The result was considered valid when at least two doctors agreed. Otherwise, a re-evaluation will be conducted until a consensus is reached by two or more people.

Adverse reactions occurring during and after HMME-PDT were recorded, primarily including local skin reactions (edema, burning, scabbing, pigmentation, scar, atrophy, and ulcer) and systemic adverse reactions.

A descriptive summary of demographic characteristics was provided, presenting categorical variables as counts and percentages. Chi-square tests and Fisher’s exact probability tests were used to explore associations between potential prognostic factors and response. Multivariate logistic regression models were further employed to identify correlations and assess independent factors influencing treatment outcomes. Results were expressed as odds ratios (OR) with corresponding 95% confidence intervals (CI). *p*-values <0.05 were considered statistically significant. Statistical analysis was performed using SPSS (version 24.0, IBM INC, Illinois, United States).

## Results

### Patient baseline data

Data were collected from a total of 152 patients, among whom 12 were lost to follow-up or had no evaluable photographs. The final study included 140 patients meeting the inclusion criteria, comprising 78 female and 62 male patients, with a mean age of 7.61 years (range: 1–18 years). There were 93 cases with pink lesions and 47 with purple lesions. Regarding lesion locations, 53 patients had lesions in the midface, 60 in the lateral face, 5 on the scalp, 9 on the neck, and 13 on the extremities. in total, 94 patients had a history of PDL treatment, while 36 had no prior PDL treatment. Patient demographics and treatment histories are summarized in Table 1 (by age group).

**Table 1:** Basic characteristics of the patients before treatment.

Characteristic	1–6 years old (*n* = 42)	6–12 years old (*n* = 68)	12–18 years old (*n* = 30)	Total (*n* = 140)
Gender				
Males	20 (47.6%)	31 (45.6%)	11 (36.7%)	62 (44.3%)
Females	22 (52.4%)	37 (54.4%)	19 (63.3%)	78 (55.7%)
Type of lesion				
Red	30 (71.4%)	42 (61.8%)	21 (70.0%)	93 (66.4%)
Purple	12 (28.6%)	26 (38.2%)	9 (30.0%)	47 (33.6%)
Lesion location				
Mid-face	16 (38.1%)	27 (40.0%)	10 (33.3%)	53 (37.9%)
Periphery of the face	21 (50.0%)	28 (41.2%)	11 (36.7%)	60 (42.9%)
Scalp	1 (2.4%)	1 (1.5%)	3 (10%)	5 (3.6%)
Neck	1 (2.4%)	5 (7.4%)	3 (10%)	9 (6.4%)
Limbs	3 (7.1%)	7 (10.3%)	3 (10%)	13 (9.3%)
Lesion area				
<30 cm^2^	29 (69.0%)	43 (63.2%)	24 (80%)	96 (68.6%)
≥30 cm^2^	13 (31.0%)	25 (36.8%)	6 (20%)	44 (31.4%)
Treatment interval				
<6 months	26 (61.9%)	36 (52.9%)	13 (43.3%)	75 (53.6%)
≥6 months	16 (38.1%)	32 (47.1%)	17 (56.6%)	65 (46.4%)
History of PDL therapy				
Yes	27 (64.3%)	44 (64.7%)	23 (76.7%)	94 (67.1%)
No	15 (35.7%)	24 (35.3%)	7 (23.3%)	46 (32.9%)

### Efficacy

**Figure 1:**
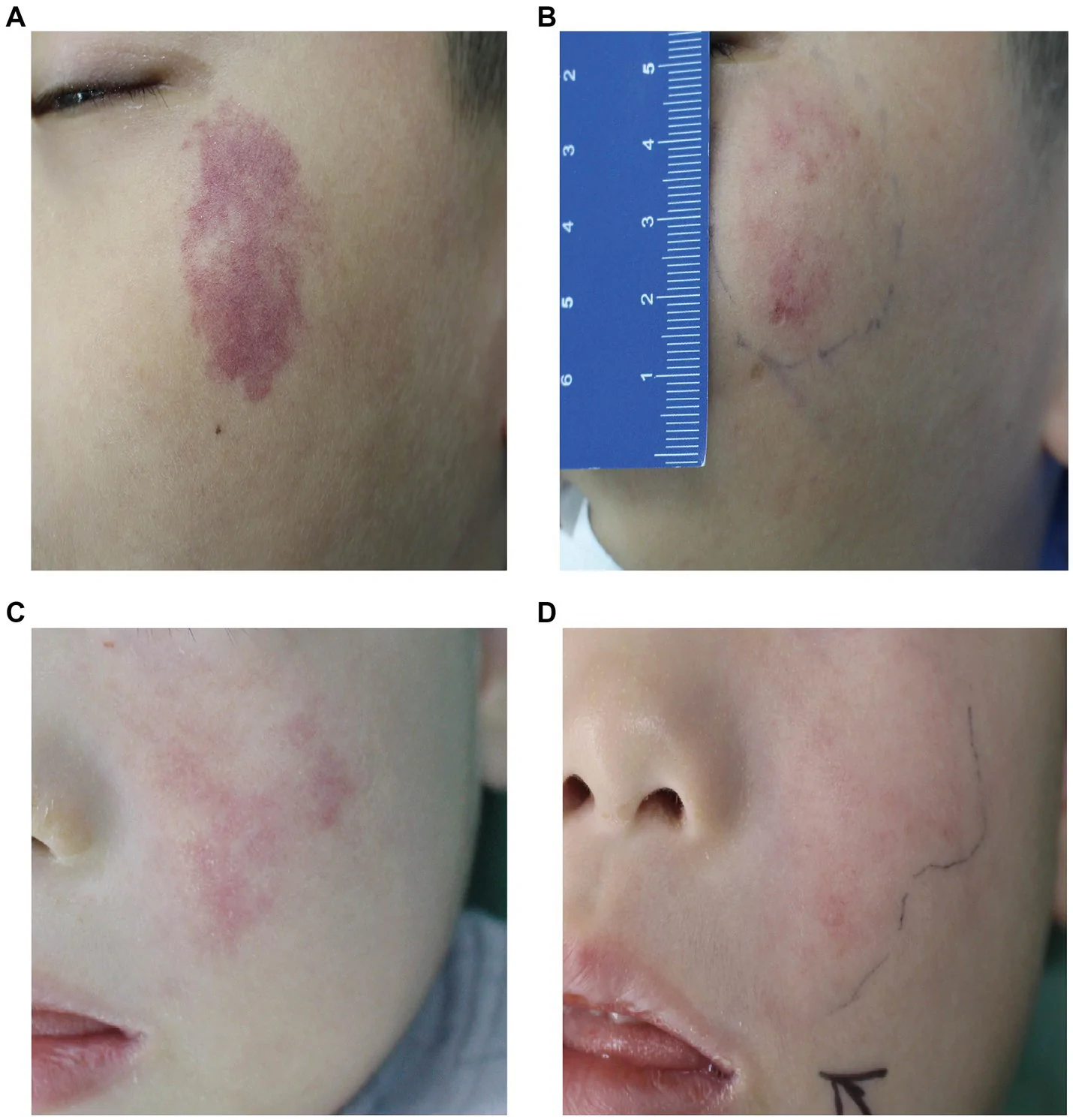
Photographs of patients before and after the second session of HMME-PDT. Red-type PWS: **A,B**; Purple-type PWS: **C,D**.

After two HMME-PDT sessions, 33 patients achieved a cure (27 in the red group, 6 in the purple group), accounting for 23.6%. A total of 39 patients demonstrated marked improvement (29 in the red group and 10 in the purple group), representing 27.9%. In total, 39 patients showed moderate improvement (24 in the red group, 15 in the purple group), also accounting for 27.9%. A total of 28 patients showed partial response (red group: 13; purple group: 15), accounting for 20.0%, and 1 patient showed no response (red group: 0; purple group: 1), accounting for 0.7% (Table 2). The efficacy rate of 86.0% in the red group was significantly higher than that of 66.0% in the purple group. The difference in therapeutic efficacy between the two lesion colors was statistically significant (*p* = 0.013). Figure 1 shows representative photos of a PWS patient before treatment and after two treatments.

**Table 2:** Response after the second session of HMME-PDT.

Factors	Class of response					*p*-value
	Excellent	Good	Fair	Poor	No	
Age						
1–6 years old	14	15	10	3	/	0.046
6–12 years old	13	20	20	14	1	
12–18 years old	6	4	9	11	/	
Gender						
Males	13	17	18	13	1	0.883
Females	20	22	21	15	/	
Type of lesion						
Red	27	29	24	13	/	0.013
Purple	6	10	15	15	1	
Lesion location						
Mid-face	16	15	12	9	1	0.034
Periphery of the face	15	21	17	7	/	
Scalp	1	1	2	1	/	
Neck	0	1	4	4	/	
Limbs	1	1	4	7	/	
Lesion area						
<30 cm^2^	23	32	24	17	/	0.111
≥30 cm^2^	10	7	15	11	1	
Treatment interval						
<6 months	19	25	24	7	/	0.007
≥6 months	14	14	15	21	1	
History of PDL therapy						
Yes	25	27	22	20	/	0.223
No	8	12	17	8	1	

Additionally, significant differences were observed in treatment efficacy based on lesion location, age group, and whether the treatment interval was 6 months (*p* = 0.013, 0.034, 0.046, and 0.007, respectively). However, no statistically significant differences were observed in treatment efficacy based on gender, prior PDL treatment history, or lesion area exceeding 30 cm^2^ (*p* = 0.883, *p* = 0.223, *p* = 0.111, respectively).

We incorporated lesion color, lesion location, age group, and treatment interval (whether 6 months) into a multivariate logistic regression model. Results showed that children aged 1–6 years demonstrated superior treatment efficacy compared to those over 12 years (OR = 0.285, 95% CI: 0.116–0.698, *p* = 0.006), while no statistically significant difference existed between children aged 6–12 years and those aged 12–18 years (*p* > 0.05). Treatment outcomes for red lesions were superior to those for purple lesions (OR = 0.361, 95% CI: 0.182–0.715, *p* = 0.003). Lesions in the midface and lateral face showed better outcomes than those on the extremities (OR = 0.177, 95% CI: 0.054–0.581, *p* = 0.004) and (OR = 0.165, 95% CI: 0.051–0.539, p = 0.003). Treatment intervals exceeding 6 months did not affect efficacy (*p* > 0.05) (Table 3).

**Table 3:** Multivariate logistic regression analysis on the response rate of the second session of HMME-PDT.

Factors	β	SE	Wald Chi^2^	*p*-value	OR	95% CI
Age						
1–6 years old	−1.256	0.460	7.465	0.006	0.285	0.116, 0.698
6–12 years old	−0.573	0.415	1.908	0.167	0.564	0.251, 1.271
12–18 years old	-	-	-	-	-	-
Type of lesion						
Red	−1.020	0.349	8.534	0.003	0.361	0.182, 0.715
Purple	-	-	-	-	-	-
Lesion location						
Mid-face	−1.734	0.608	8.143	0.004	0.177	0.054, 0.581
Periphery of the face	−1.802	0.604	8.897	0.003	0.165	0.051, 0.539
Scalp	−1.103	0.998	1.221	0.269	0.332	0.047, 2.347
Neck	−0.520	0.841	0.382	0.537	0.595	0.115, 3.089
Limbs	-	-	-	-	-	-
Treatment interval						
<6 months	−0.426	0.332	1.647	0.199	0.653	0.341, 1.252
≥6 months	-	-	-	-	-	-

### Adverse reactions

All patients experienced varying degrees of localized edema following HMME-PDT, which resolved spontaneously within 7 days at the latest. Most patients experienced a burning sensation post-treatment, which improved rapidly with cold sprays and ice packs. In total, 8 patients (5.7%) developed localized thin crusts that spontaneously shed without specific intervention. Five patients (3.6%) exhibited hyperpigmentation during follow-up. No cases of atrophy, ulceration, scarring, or other treatment-related adverse effects were observed. No systemic adverse events were reported.

## Discussion

PWS typically affects the face, leading to cosmetic concerns, significant psychological distress, and reduced quality of life (6, 8, 14, 15). Public awareness of PWS also drives a preference for early intervention (16). While PDL is currently considered the gold standard for treating PWS (5, 9), its efficacy remains limited. In recent years, concerns over technical instability and high financial impact due to system maintenance and mandatory dye kits have increased interest in alternative treatment approaches (17).

HMME-PDT is a novel therapy increasingly used for treating PWS due to its significant efficacy and good tolerability. However, its package insert still recommends use only in patients aged 14 years and older. Previous studies have predominantly focused on adolescent and adult patients (16, 18), with few reports specifically addressing pediatric patients (15). In contrast, all subjects in our study were under 18 years of age.

We conducted a promising analysis of 140 pediatric cases and found that the efficacy rate was significantly higher in children with red lesions than in those with purple lesions, consistent with the findings of several studies (15, 19, 20). Ling Wang believes that different types of PWS may exhibit distinct vascular structures, vascular densities, and skin tissue densities (21). These parameters influence light penetration and the intensity of photochemical reactions. Liu’s research found that as PWS progress from red to purple lesions, the diameter of blood vessels gradually increases, the vessel walls thicken, and the vessels deepen in location (22). The penetration depth of 532 nm lasers is limited, approximately 1–2 mm, making it difficult to effectively activate photosensitizers within deep-seated blood vessels (23, 24). Moreover, the faster blood flow velocity within deep large vessels shortens the residence time of HMME and reduces the photochemical reaction window period (25), which is also one reason for the poor treatment outcomes in purple lesions.

In this study, we also found that patients younger than 6 years of age demonstrated better treatment outcomes than those older than 12 years. Children with PWS initially present with red patches, which gradually darken to purple with age and may even develop into proliferative nodules. Many studies have reported that younger patients achieve better outcomes (16, 19, 26). A possible reason is that in younger children, the epidermis is thinner and the affected blood vessels are located more superficially. This allows the laser to penetrate the skin more easily and be absorbed by the oxygenated hemoglobin in the blood vessels, resulting in a more potent therapeutic effect (22). With the rise of science popularization content creators, many people learn about the characteristics of PWS through short video platforms and choose to seek treatment during childhood. Our study included pediatric patients and did not involve cases with thickened nodular lesions, so we cannot draw conclusions regarding those specific presentations. Nevertheless, we still recommend that patients seek treatment as early as possible to achieve better outcomes and potentially reduce the psychological impact of appearance on a child’s development.

Our research has found that lesions in the midface and lateral face respond better to treatment than those in the extremities, consistent with findings from certain studies (15, 16, 26). A clinical study indicates that lesions located on the forearm and fingers show no significant improvement following treatment (18). Some studies suggest that facial skin is thinner (average epidermis-dermis thickness of 1.5–2 mm), allowing light energy to more readily reach target blood vessels. In contrast, skin on the extremities is thicker (>3 mm) and has a more developed stratum corneum, which may reduce light-absorption efficiency (20, 27). The facial skin is characterized by a rich blood supply and an extensive subepidermal capillary network. The capillary density in the trigeminal nerve distribution area of the face (V1-V2) is significantly higher than in the extremities (28). During PDT, the greater the accumulation of singlet oxygen in capillary walls, the more effective the treatment may be for facial lesions compared to those on the limbs (29). Therefore, despite the limited evidence-based data on PDT, existing evidence suggests that the anatomical advantages of the face may stem from: (1) more superficial and densely distributed vascular plexuses; (2) optimized light transmission due to thin skin; and (3) region-specific neurovascular regulatory mechanisms (28, 30).

Multivariate logistic regression found that previous PDL treatment history had no significant impact on treatment efficacy. Numerous studies have also demonstrated that HMME-PDT is effective for patients who have failed PDL therapy (16, 31, 32). The core mechanism of PDT involves photosensitizers (such as HMME) specifically accumulating within vascular endothelial cells. Upon exposure to light of a specific wavelength, these photosensitizers generate singlet oxygen, which directly damages the endothelial cells rather than relying on hemoglobin to absorb heat (as in pulsed dye lasers) (23). Previous PDL treatment may cause superficial vascular fibrosis or thrombosis, but residual deep abnormal vessels retain intact endothelial structures, allowing HMME to accumulate in these non-occluded vessels and exert its effects. Clinical data show no statistical difference in the clearance rate of deep malformed vessels between patients with previous PDL treatment switching to PDT and treatment-naïve patients (23, 33). Therefore, we believe that a history of PDL therapy does not affect the efficacy of hemoporfin PDT.

Modern clinical practice increasingly emphasizes individualized treatment while adhering to established therapeutic principles. Identifying factors that influence therapeutic efficacy is a prerequisite for such individualized approaches. In glioblastoma, radiomics models that integrate imaging and biological information have been shown to enable patient risk stratification (34). In the present study, multivariate analysis identified age under six years, red lesion type, and facial involvement as independent predictors of response to hemoporfin photodynamic therapy, thereby providing actionable indicators to guide clinical decision-making.

Regarding adverse reactions, we observed edema in nearly all patients, with its severity positively correlated with the photosensitizer dose and light intensity (23, 35). However, in most cases, it was transient and self-limiting. This is similar to some studies (16, 35). Moreover, PDT has minimal impact on normal epidermis, possibly due to the vascular targeting mechanism of PDT (36), and also benefits from the application of protective coverings over unaffected skin areas. All patients undergo treatment under general anesthesia, with no pain responses during surgery. Postoperatively, younger children often exhibit irritability and crying. We believe pain is one contributing factor, while cardiac monitoring and oxygen administration may also cause discomfort. A small number of pediatric patients developed crusting and hyperpigmentation, all of which resolved spontaneously within 3 months following treatment. Of note, children presenting with crusting should be closely monitored to prevent secondary infection, as uncontrolled infection may result in scar formation. Therefore, we conducted preoperative education for guardians, including detailed information on the treatment process, strict sun protection requirements, and the optional use of antimicrobial agents when necessary. These interventions helped reduce the incidence of adverse reactions and improve overall patient satisfaction. Additionally, pediatric patients require vigilance against the risk of photosensitivity reactions. Safety can be further enhanced through parameter optimization, such as reducing light power density, employing cold spray cooling, and implementing moist wound healing care (23). Regarding systemic safety, no adverse events involving internal organs (e.g., hepatic, renal, or hematologic systems) were reported during the study period. Nevertheless, we agree that future studies should incorporate routine laboratory monitoring, including liver and kidney function tests, to comprehensively evaluate systemic safety, especially in younger children. In summary, we believe that HMME-PDT is safe and feasible for children with PWS older than 1 year.

We acknowledge that the requirement for general anesthesia in all pediatric patients may limit the accessibility of this treatment in settings without anesthesia support. However, in our center, general anesthesia was necessary to ensure immobility and avoid accidental movement during laser irradiation, which could compromise efficacy or cause injury. Future studies may explore the feasibility of deep sedation or immobilization devices as alternative strategies, but until such approaches are proven safe and effective in children, we maintain that general anesthesia remains the preferred standard for maximizing procedural safety and treatment outcomes.

Although PDT can improve erythematous manifestations in many patients, some individuals still experience suboptimal treatment outcomes. Current research in PDT is advancing in several promising directions, including the development of novel photosensitizers, as well as innovative nanocarriers and delivery systems (37, 38). There is considerable anticipation among researchers that further fundamental studies will ultimately help achieve better clinical outcomes for patients with PWS.

In this study, all patients received at least two sessions of hematoporphyrin monomethyl ether photodynamic therapy (HMME-PDT). After two sessions, 29 patients still exhibited suboptimal responses. Among them, 13 patients underwent a third session. Preliminary observations showed that the third session provided additional improvement in 8 of 13 patients (61.5%). However, given the limited sample size and the lack of standardized retreatment criteria, the benefit of more than two sessions requires further validation in future studies.

This study has several limitations. First, this study did not include patients under one year of age, thus no efficacy and safety data for children under one year old were obtained. Second, although this was a prospective study, the absence of a control group precluded direct comparisons with other groups (e.g., PDL or placebo groups), and direct comparisons between the present intervention and other therapeutic modalities could not be performed. Future prospective comparative cohort studies are warranted to validate these findings. Third, this study did not formally calculate inter-rater reliability (e.g., Fleiss’kappa) for the subjective efficacy grading. Although three blinded dermatologists independently assessed the photographs and a majority rule was applied, the lack of a reported agreement coefficient may introduce potential bias. Future studies should incorporate validated objective instruments such as VISIA or OCT, and report inter-rater reliability. We expect to conduct large-scale, long-term prospective studies to verify the efficacy and safety of HMME-PDT and explore other factors influencing the treatment outcomes.

## Conclusion

HMME-PDT is a safe and effective treatment for PWS in children. It demonstrates superior efficacy in children aged 1–6 years, for pink lesions and facial areas. We also recommend earlier initiation of HMME-PDT therapy to achieve greater improvement.

## Data Availability

The raw data supporting the conclusions of this article will be made available by the authors, without undue reservation.

## References

[1] Tian R Wang Q Li S Nong X. Non-invasive efficacy assessment of pulsed dye laser and photodynamic therapy for port-wine stain. Indian J Dermatol Venereol Leprol. (2024) 90:615–22. doi: 10.25259/IJDVL_985_2023, 38841964

[2] Lederhandler MH Pomerantz H Orbuch D Geronemus RG. Treating pediatric port-wine stains in aesthetics. Clin Dermatol. (2022) 40:11–8. doi: 10.1016/j.clindermatol.2021.08.006, 35190059

[3] Mohamed EM Abdel-Aleem HL Mansour M Rageh MA. Efficacy and prognostic factors for successful treatment of port-wine stains by 577-nm yellow laser: a cohort study on 42 patients. Lasers Med Sci. (2025) 40:90. doi: 10.1007/s10103-025-04350-w, 39951218 PMC11828787

[4] Chen JK Ghasri P Aguilar G Van Drooge AM Wolkerstorfer A Kelly KM . An overview of clinical and experimental treatment modalities for port wine stains. J Am Acad Dermatol. (2012) 67:289–304.e29. doi: 10.1016/j.jaad.2011.11.938, 22305042 PMC4143189

[5] Van Drooge AM Beek JF Van Der Veen JP Van Der Horst CM Wolkerstorfer A. Hypertrophy in port-wine stains: prevalence and patient characteristics in a large patient cohort. J Am Acad Dermatol. (2012) 67:1214–9. doi: 10.1016/j.jaad.2012.05.027, 22749320

[6] Nguyen L Kleeberg A Seeber N Kimmig W Schneider SW Herberger K. Pulsed dye laser treatment of port-wine stains—analysis of health service in Germany. J German Soc Dermatol. (2023) 21:1218–20. doi: 10.1111/ddg.15181_g, 37550854

[7] Hagen SL Grey KR Korta DZ Kelly KM. Quality of life in adults with facial port-wine stains. J Am Acad Dermatol. (2017) 76:695–702. doi: 10.1016/j.jaad.2016.10.039, 27955934 PMC5790427

[8] Chou M Karim M Josephs J Itzkowitz T Dreker MR Labadie JG. Pulsed dye laser and adjuvant topical therapies for the treatment of port-wine stains: a systematic review. Lasers Surg Med. (2024) 56:39–44. doi: 10.1002/lsm.23706, 37431532

[9] Van Raath MI Bambach CA Dijksman LM Wolkerstorfer A Heger M. Prospective analysis of the port-wine stain patient population in the Netherlands in light of novel treatment modalities. J Cosmet Laser Ther. (2018) 20:77–84. doi: 10.1080/14764172.2017.1368669, 29384394

[10] Gao K Huang Z Yuan KH Zhang B Hu ZQ. Side-by-side comparison of photodynamic therapy and pulsed-dye laser treatment of port-wine stain birthmarks. Br J Dermatol. (2013) 168:1040–6. doi: 10.1111/bjd.12130, 23137063

[11] Wu Q Tu P Zhou G Yang H Zhou Z Zhao Y . A dose-finding study for Hemoporfin in photodynamic therapy for port-wine stain: a multicenter randomized double-blind phase Iib trial. Photodermatol Photoimmunol Photomed. (2018) 34:314–21. doi: 10.1111/phpp.12384, 29533491

[12] Zhu Y Ding C Fang W Li T Yan L Tian Y . Metal-polyphenol self-assembled Nanodots for NIR-II fluorescence imaging-guided Chemodynamic/photodynamic therapy-amplified Ferroptosis. Acta Biomater. (2024) 185:361–70. doi: 10.1016/j.actbio.2024.07.017 39025392

[13] Zhao Y Tu P Zhou G Zhou Z Lin X Yang H . Hemoporfin photodynamic therapy for port-wine stain: a randomized controlled trial. PLoS One. (2016) 11:E0156219. doi: 10.1371/journal.pone.0156219, 27227544 PMC4881994

[14] You R Tan Y Zhang X Li L Zeng T Zhou Y . Influential factors in the efficacy and safety of Hemoporfin-mediated photodynamic therapy for facial port-wine stains. J Cosmet Dermatol. (2025) 24:E70153. doi: 10.1111/jocd.70153, 40165716 PMC11959330

[15] Li-Qiang G Hua W Si-Li N Chun-Hua T. A clinical study of HMME-PDT therapy in Chinese pediatric patients with port-wine stain. Photodiagn Photodyn Ther. (2018) 23:102–5. doi: 10.1016/j.pdpdt.2018.06.006, 29885812

[16] Zhang X Yuan C Xiao X Yin R Lei H Li Y . Hemoporfin-mediated photodynamic therapy for the treatment of port-wine stain: a multicenter, retrospective study. Photodiagnosis Photodyn Ther. (2023) 42:103545. doi: 10.1016/j.pdpdt.2023.103545, 37001715

[17] Nguyen L Sorbe C Schoen G Schneider SW Herberger K. Laser and light-based treatments for port-wine birthmarks—a systematic review and network Meta-analysis. J German Soc Dermatol. (2025) 23:293–301. doi: 10.1111/ddg.15612, 39668417 PMC11887003

[18] Zhang Y Zou X Chen H Yang Y Lin H Guo X. Clinical study on clinical operation and post-treatment reactions of HMME-PDT in treatment of PWS. Photodiagn Photodyn Ther. (2017) 20:253–6. doi: 10.1016/j.pdpdt.2017.09.013, 29079350

[19] Huang Y Yang J Sun L Zhang L Bi M. Efficacy of influential factors in Hemoporfin-mediated photodynamic therapy for facial port-wine stains. J Dermatol. (2021) 48:1700–8. doi: 10.1111/1346-8138.16094, 34355416

[20] Chun-Hua T Li-Qiang G Hua W Jian Z Si-Li N Li L . Efficacy and safety of Hemoporfin photodynamic therapy for port-wine stains in Paediatric patients: a retrospective study of 439 cases at a single Centre. Photodiagn Photodyn Ther. (2021) 36:102568. doi: 10.1016/j.pdpdt.2021.102568 34614424

[21] Wang L Li L Huang C. Efficacy of photodynamic therapy in the treatment of port wine stains: a systematic review and Meta-analysis. Front Med. (2023) 10:1111234. doi: 10.3389/fmed.2023.1111234, 36895715 PMC9988944

[22] Liu L Zhou L Zhao Q Li X Yang L Li E . Histological analysis of different types of port-wine stains to guide clinical decision making: a retrospective study. Indian J Dermatol Venereol Leprol. (2023) 89:204–12. doi: 10.25259/IJDVL_730_2021, 35593279

[23] Khalaf AT Wei Y Abdalla AN Fan W Jiang H. Recent Progress in Hematoporphyrin monomethyl ether-photodynamic therapy for port-wine stains: updates and insights. Arch Dermatol Res. (2024) 317:28. doi: 10.1007/s00403-024-03531-x, 39549139

[24] Guo Z Zhou W Ke C Huang Z Wang Y Zhu Y . Simulating PDT of port-wine stains in the in vivo chicken wattle model using Hemoporfin and radiation at 532 nm: comparison of a LED and a laser source. Photodiagn Photodyn Ther. (2024) 46:104068. doi: 10.1016/j.pdpdt.2024.104068, 38598961

[25] Li ZH Meng DS Li YY Lu LC Yu CP Zhang Q . Hypericin damages the Ectatic capillaries in a Roman cockscomb model and inhibits the growth of human endothelial cells more potently than Hematoporphyrin does through induction of apoptosis. Photochem Photobiol. (2014) 90:1368–75. doi: 10.1111/php.12323, 25065502

[26] Lin Y Gong W Kang J Fang Y Liu J Lin L . Hemoporfin-mediated photodynamic therapy for port-wine stains: multivariate analysis of clinical efficacy and optical coherence tomography appearance. Front Med. (2022) 9:800836. doi: 10.3389/fmed.2022.800836, 35280862 PMC8908093

[27] Mehrabi JN Holmes J Abrouk M Wang JV Pomerantz H Palma AM . Vascular characteristics of port wine birthmarks as measured by dynamic optical coherence tomography. J Am Acad Dermatol. (2021) 85:1537–43. doi: 10.1016/j.jaad.2021.08.007, 34390783

[28] Chen Y Liu H Zhou J Yang X Jia H Ma G . Genotypes and phenotypes of capillary malformation-arteriovenous malformation: characterization and correlation analysis. Int J Dermatol. (2025) 64:693–701. doi: 10.1111/ijd.17504, 39367533

[29] Hu XM He L Zhao Y. Dynamic model of vascular-targeted photodynamic therapy. Comput Methods Biomech Biomed Engin. (2017) 20:1056–65. doi: 10.1080/10255842.2017.1331344, 28521517

[30] Dingenen E Segers D De Maeseneer H Van Gysel D. Sturge-weber syndrome: an update for the pediatrician. World J Pediatrics. (2024) 20:435–43. doi: 10.1007/s12519-024-00809-y, 38658498

[31] Zhang LC Yang J Huang YB Bi MY. Efficacy of Hemoporfin photodynamic therapy for pulsed dye laser-resistant facial port-wine stains in 107 children: a retrospective study. Indian J Dermatol Venereol Leprol. (2022) 88:275. doi: 10.25259/IJDVL_976_20, 34672476

[32] Zhang M Wu Q Lin T Guo L Ge Y Zeng R . Hematoporphyrin monomethyl ether photodynamic therapy for the treatment of facial port-wine stains resistant to pulsed dye laser. Photodiagn Photodyn Ther. (2020) 31:101820. doi: 10.1016/j.pdpdt.2020.101820, 32428574

[33] Qiu H Gu Y Wang Y Huang N. Twenty years of clinical experience with a new modality of vascular-targeted photodynamic therapy for port wine stains. Dermatol Surg. (2011) 37:1603–10. doi: 10.1111/j.1524-4725.2011.02129.x, 21883647

[34] Wang X Yang M Li J Wu Y Yue Q Wang X . Treg-driven magnetic resonance Radiomics in glioblastoma: a multicenter and cross-species model for prognostic biomarker discovery. Cancer Lett. (2026) 642:218303. doi: 10.1016/j.canlet.2026.218303, 41655941

[35] Diao P Han C Li X Yang Y Jiang X. Hematoporphyrin monomethyl ether photodynamic therapy of port wine stain: narrative review. Clin Cosmet Investig Dermatol. (2023) Volume 16:1135–44. doi: 10.2147/CCID.S401447, 37139084 PMC10150768

[36] Pu Y Chen W Yu Z. Research Progress of Hemoporfin—part one: preclinical study. Photodiagn Photodyn Ther. (2012) 9:180–5. doi: 10.1016/j.pdpdt.2011.09.004, 22594989

[37] Huang R Zhou P Chen B Zhu Y Chen X Min Y. Stimuli-responsive Nanoadjuvant rejuvenates robust immune responses to sensitize Cancer immunotherapy. ACS Nano. (2023) 17:21455–69. doi: 10.1021/acsnano.3c06233, 37897704

[38] Wu T Wei P Zhao P Niu X Ding C YJCEJ Z. Engineering local coordination environment of manganese single-atom enzyme for amplified infected wound therapy. Chem Eng J. (2025) 514:163247. doi: 10.1016/j.cej.2025.163247, 38826717

